# Impact of arc quenching parameters on surface hardness and microstructure of S45C steel with concave surfaces

**DOI:** 10.1371/journal.pone.0324922

**Published:** 2025-06-02

**Authors:** Van-Thuc Nguyen, Dang Thu Thi Phan, Huynh Do Song Toan, Tran Thai Son, Pham Son Minh, Nguyen Ho

**Affiliations:** 1 HCMC University of Technology and Education, Ho Chi Minh City, Vietnam; 2 Faculty of Mechanical Engineering, HCMC University of Technology and Education, Ho Chi Minh City, Vietnam; UNICAMP, University of Campinas, BRAZIL

## Abstract

This study investigates the effects of arc length, current intensity, travel speed, gas flow rate, and pulse time on surface hardness to better understand the arc quenching of S45C steel with a curved shape. With the standard examination method, increasing the current intensity, Travel speed, and arc length causes the surface hardness to decrease. The surface hardness varies depending on the gas flow rate and pulse time. The Travel speed factor appears to have the greatest effect, followed by the gas flow rate and current intensity. Pulse time and arc length are ranked fourth and fifth, respectively, indicating a smaller impact on surface hardness. The microhardness diagram is divided into four stages: improving, rapid dropping, moderate dropping, and stable. The greatest hardness was 576 HV, with a case depth of 1200 μm. The structure of the arc-hardened sample is composed of hardening zones, HAZ, and base metal. The base metal is composed of ferrite and pearlite, which are the original microstructures of medium-carbon steel. The HAZ is made up of two phases: a brown bainite phase and a brighter ferrite phase. Ferrite, bainite, martensite, and residual austenite phases make up the hardening with a high hardness value area. These phases’ diversity results from their rapid heating and cooling rates as well as the significant variations in cooling rates among depths. The findings of the study on the optimum values of factors such as current intensity at 150 A, Travel speed at 150 mm/min, arc length at 2.5 mm, pulse time at 0.6 s, or gas flow at 10.5 l/min can help engineers to have a closer look at the parameters of this arc tempering technology affecting the surface hardness and their applications. Moreover, the hardness measurement value according to the Taguchi method investigation also shows that the highest value of surface hardness achieved is 42.6 HRC compared to 18 HRC of unhardened surface hardness. In addition, the findings on microstructure also help the applicator to better understand and evaluate the quality of this electric arc method for quenching the surface of S45C steel, thereby making it more useful in the industry.

## Introduction

Traditional quenching and tempering heat treatments have been utilized for centuries on steels to achieve favorable combinations of strength and toughness from the martensitic structure. In addition, heat treatment of the material is aimed at improving its mechanical properties and anisotropy. Manuscript of Jairo Alberto.M et al.[1] examined the effects of different heat treatments on the mechanical behavior, anisotropy, and microstructure of a sub-eutectic, near-eutectic AlSi11Cu alloy obtained by laser powder melting (L-PBF). Different strength-ductility ratios were obtained after treatment at different temperatures (200°C–550°C). The study also showed that heat treatment at temperatures up to 400°C or higher, followed by water quenching, resulted in good strength-to-ductility ratios while reducing anisotropy. Besides volumetric quenching for the whole body, the surface quenching for the local area provides a thin hardening layer for the specific location [2–4]. Applying the surface quenching could save energy, reduce volumetric torsion, and decarbonize while preserving the toughness of the not-heated areas. The surface quenching technique could be applied using many types of heat generations, such as induction heating, laser heating, plasma heating, flame, electron beam, and arc quenching techniques [5–7].

Induction and flame hardening can be classified as two-step hardening as they require heating and quenching [8]. Induction hardening is a method of surface quenching that involves induction-heating the surface of magnetic materials following a quenching procedure. This hardening process has many advantages: high precision, good hardening speed, low rate of distortion, and high energy efficiency [9]. However, this technique also has disadvantages, such as the high initial setup cost, operational difficulty, and only application to ferrous metals [10]. Gao et al. [11] investigated the impact of induction hardening on railway axles, focusing on the damage tolerance. An artificial impact damage is created in the hardened sample. The induction-hardened samples have an improvement of 61% in the fatigue strength compared to the untreated sample. This improvement is due to the generation of a high-strength hardened layer with compressive residual stress. With moderate impact, induction-hardened samples present a better plastic deformation resistance. However, the hardened samples must suffer microcracks and material loss under ballistic impact.

To evaluate more closely, Maialen Areitioaurtena et al. [8] used finite element modeling to simulate and experimentally investigate residual stresses during induction quenching of 42CrMo4 steel. The induction quenching process generates compressive residual stresses in the martensite layer of the heat-treated surface and tensile stresses in the untreated core. The residual stresses generated by the induction quenching process have a significant impact on fatigue performance because they act as crack growth retardants. Kattimani et al. [12] examined the fatigue characteristics of the IN718 alloy gas turbine after conducting the induction hardening process. Gas turbines work under high-stress conditions; therefore, the fatigue strength is a critical requirement. The samples are heated at 850°C and 1000°C, following an oil quenching step. The fracture mode of the IN718 sample is transgranular. At both room temperature and 800°C, the heat-treated samples present much higher fatigue strength than the untreated ones. Therefore, the fatigue life and safety of the gas turbine components are greatly improved.

Flame hardening is a more traditional hardening method, using a high-temperature flame to heat the components and then cool them. Besides some similar merits and demerits as the induction hardening technique, the flame hardening technique has more concerns about heat control and negative environmental impact. Thamilarasan et al. [13] applied the Taguchi method to optimize the microhardness of the flame-hardening process of low-carbon steel. Three parameters, including flame temperature, torch cap, and quenching time, are surveyed. The results indicated that the quenching period plays the most important role in the microhardness, followed by the flame temperature and torch cap. Moreover, the optimization process could improve the microhardness to 700 HV at a quenching time of 40 s, a flame temperature of 1000°C, and a torch cap of 35 mm.

Different from these above hardening methods, which require a further quenching stage after heating, some advanced methods could produce a thin hardening layer without quenching. These methods, such as laser, electron beam, plasma, and arc hardening, apply stronger heating generators in a shorter time. Therefore, the heated surface is cooled down by the core of the components, called self-quenching, creating a thin martensite layer. The application of laser energy to temper steel surfaces to improve hardness, wear resistance, and increase impact resistance has been widely studied [14]. Muthukumaran et al. [15] compared the effects of laser hardening with different laser types and steel grades. They applied CO- laser, Nd: YAG laser, fiber laser, and diode laser to increase the temperature of many steel grades. Despite changing the laser types, heating parameters, and steel grades, the hardness difference in the single-track hardening and multi-track hardening is significant due to the overlapped heating region. In the overlapped region, the steel is strongly tempered due to the back-tempering phenomenon. Lakhkar et al. [16] also investigated the multi-track phenomenon of the laser hardening process by using the tempering modeling method. The model could help increase the uniformity and case depth of the large surface. This model is built to predict the hardening process, case depth value, and back-tempering phenomenon by mitigating the three-dimensional transient temperature and kinetic hardening–tempering models. The model is also validated by multi-track laser hardening testing in AISI 4140 steel. The laser optimization case depth can reach 2 mm by selecting the track overlapped by 5 mm, creating a good uniform hardness along the track.

The method of using a heat source from an electron beam to harden the surface is an alternative solution to the above methods. Basak et al. [17] improved the wear and corrosion resistance of 316L steel by using the electron beam hardening method. Interestingly, the molten and then rapid solidification layer could create a dendritic structure. The surface becomes slightly rougher after the electron beam hardening process with the presence of micro ripples. Interestingly, the grain size reduces dramatically from 5 μm to 1 μm. The hardened layer possesses excellent hardness, wear, and corrosion resistance. The surface hardness improved by 22%, while the cumulative wear depth reduced by 77% compared to the untreated samples. The reason for these enhancements is the improvement of dislocation density caused by thermal stresses. Some metal carbide phases also appear in the samples after the electron beam hardening treatment, also contributing to the hardness improvement. The heat source generated from plasma energy for surface quenching is also widely used in industry. Sagdoldina et al. [18] improve the surface hardness of 40 Kh steel by plasma hardening. The case depth is 1.6 mm, while the hardness could be 966 HV. The surface structure is transformed from a ferrite and pearlite structure to a martensite structure due to self-quenching. Laser or electron beam setup costs are high due to their complex and state-of-the-art structures.

Arc hardening appears to be much cheaper due to the availability of the arc source via arc welding equipment. According to Safonov et al. [19], the arc quenching process can achieve a maximum hardness of 50–60 HRC with a case depth of 1.5-2.0 mm, depending on the steel grades and the processing parameters such as arc current and travel speed. The findings demonstrate that the hardening zone’s cross-section exhibits the characteristic sickle contour. In the area close to the zone center, the surface has the highest hardness. In this study, the surface layer hardness was 240–260 HB after arc surfacing with Np-30KhGSA wire in three layers and tempering to remove tensions and 380–420 HB after turning and electric arc quenching. The roll’s wear resistance after use was comparable to that of a new, non-hardened roll made of 60KhN steel. The study of Mikheev et al. [20] investigated two parameters of the surface of medium carbon steel quenched by electric arc energy: the travel speed and the current intensity. The travel speed increases, and the thickness of the hardened zone will decrease because the amount of heat transferred from the arc tip to the surface decreases due to insufficient time to receive enough arc heat. The travel speed also affects the surface hardness; the optimal speed range was 0.06-0.09 m/s. When the current intensity increases, the depth of the hardened zone will increase, but the surface hardness will decrease. The maximum hardness achieved for steel 40 was 7.5 GPa when the current intensity was 130 A and the travel speed was 0.09 m/s. The case depth could reach a value of 300‒400 μm. The wear resistance of the arc-quenched sample has been improved four times compared to the untreated sample.

Besides the martensite phase generated during the quenching process, rapid cooling could facilitate the presence of residual austenite and bainite processes, as the study indicated by Morawiec et al. [21]. Prior martensite formation accelerated the bainite transformation, refined the microstructure, and improved the mechanical properties of the 3% Mn multiphase steel. Besides martensite, austenite, and bainite phases, Kumar et al. [22] pointed out the existence of the ferrite phase after the arc quenching process. They investigated the characteristics of AISI 8620 steel via arc quenching, focusing on the thermal and metallurgical properties. The microstructure of the arc quenching samples could be divided into three zones, including the hardening zone, the heat-affected zone (HAZ), and the base metal. They also indicated that with the presence of martensite and bainite, the wear resistance, fatigue strength, and corrosion ability of the hardened steel are enhanced. The compressive residual stress also contributed to the improvement of the mechanical properties.

Process optimization in industry has many benefits. Among them, the Taguchi method is an effective optimization tool widely applied to improve specific characteristics. For example, Saravanan et al. [23] optimized the quench-polish-quench coating process via the Taguchi method. The impact of heating temperature, nitriding time, and oxidation time on the case depth and hardness of the samples was investigated. The results revealed that the nitriding time and nitriding temperature play great roles in the case depth and hardness of the samples. Agboola et al. [24] used the Taguchi method to optimize the heat treatment parameters of medium-carbon steel. The study applied standard L9 orthogonal array experiments to survey the impact of quenchant, heating temperature, and soaking time. The results proved that soaking time is the most critical factor that impacts the hardness, yield strength, and tensile strength.

From the above studies, it can be seen that the arc energy depends on parameters such as arc length, current intensity, voltage, travel speed, and gas flow rate. In addition, the surface shape also affects the heat distribution at the surface. Unlike flat surfaces, concave and convex surfaces have different heat conduction and heat dissipation structures. In the study by Dong Ju Kim et al. [25], the authors conducted a study on heat transfer from the outside to convex, concave, and flat surfaces. This study investigated the convective heat transfer efficiency parameters of a scanning beam impinging on the surfaces according to experimental studies. The results showed that the concave surface had a better heat transfer rate from the scanning beam than the flat surface. For both concave and convex surfaces, the wall beam became thinner than the flat surface in general, which contributed to improving the heat transfer ability. The study by Bahman Meyghani and M. Awang [26] investigated the thermal and stress behavior when using friction stir welding method on two flat and curved surfaces of AA6061-T6 alloy material. The results showed that the flat surface always had a temperature 50^o^C to 80°C higher than the curved surface, and the stress of the curved surface was always lower than the flat surface when using the same input conditions. Furthermore, the area of the concave surface will be lower than the area of the convex surface below because the radius of the convex surface below will be larger than the concave surface. Therefore, compared with the same area of flat and concave surfaces, the opposite heat dissipation surface of the concave surface (convex surface) will be larger than the opposite plane of the surface to be processed. The cause of this phenomenon is lower heat penetration in the workpiece. From this study, it can be seen that the heat transfer process between the surfaces is different. The application of the arc in the process of quenching steel surface has many advantages such as easy equipment when applying with TIG arc welding equipment, faster implementation time compared to Laser due to the wider arc area, and especially the implementation cost is very low. Despite many advantages, the quenching of steel surface by electric arc has not been widely studied. In particular, the optimization of parameters in the arc quenching of steel surfaces with groove shapes has not been fully studied.

With the outstanding advantages of designing orthogonal arrays with few experiments, simultaneously evaluating many impacts of input parameters together to find optimal parameters, the optimization method using Taguchi will be used. This method will evaluate the simultaneous influence of arc quenching process parameters on the surface hardness of concave carbon steel beside the traditional method of single-variable evaluation. The arc source is generated by a tungsten internal gas (TIG) machine. The parameters affecting the arc thermal parameters, such as arc length, current intensity, travel speed, gas flow rate, and pulse time, are investigated in this paper. After quenching, the samples were evaluated by hardness testing and microstructural analysis. In parallel, the best high-hardness sample was also evaluated for surface depth hardness. The results of the investigation may provide certain insights into this arc-quenching method, the parameters affecting the quenching process, and its industrial applications.

## Experimental methods

**Fig 1:**
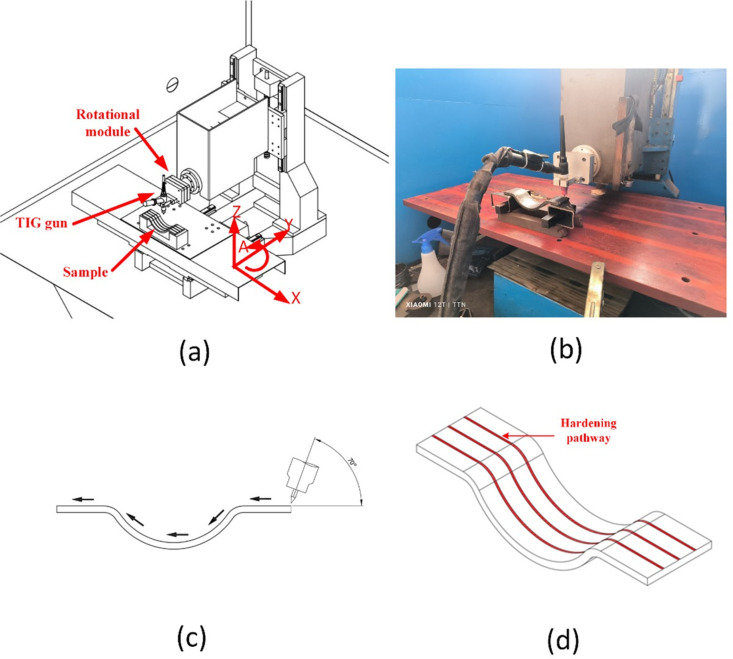
The S45C steel plate and arc quenching process: (a) S45C steel plate, (b) arc quenching machine, (c) arc quenching surface, and (d) position of hardness pathways quenching by arc energy.

The S45C steel samples used in this study have a curved shape, as detailed in Table 1. The arc tip of the TIG welding machine had a diameter of 2.5mm and was assembled on the CNC machine, as shown in Fig 1. The arc-quenching process can be controlled by changing the gas flow rate, current intensity, travel speed, arc length, and pulse time. The experimental voltage was fixed constant at 80V and the environment during the experiment was about 30°C. These parameters are investigated by the traditional method, as shown in Table 2. Besides, they are optimized by the Taguchi method, implemented by Minitab 19.1 software, as shown in Table 3. Before applying the electric arc quenching test on the surface of S45C steel, the experimental parameters (shown in Tables 2 and 3) were tested and evaluated many times for the level of deviation (tolerance). The study’s tolerance parameters were ±1 A for current intensity, ± 1 mm/min for TIG arc head travel speed, ± 0.1 mm for arc length, ± 0.2 l/min for gas flow rate, and ±0.05 s for the pulse time parameter.

**Table 1:** Chemical composition of S45C steel for the arc quenching process.

Weight %	C	Si	Mn	P	S	Ni	Cr
S45C.	0.42-0.50	0.17-0.37	0.5-0.8	0.035 max	0.035 max	0.25 max	0.25 max

**Table 2:** Experiment parameters designed by traditional method and average hardness values.

No.	Current intensity (A)	Travel speed (mm/min)	Arc length (mm)	Gas flow rate (l/min)	Pulse time (s)	HRC
1	100	200	2	10.5	0.5	41.2
2	110					38.9
3	120					37.8
4	130					37.4
5	140					36.9
6	120	150				49.6
7		175				39.4
8		200				37.9
9		225				38.4
10		250				34.9
11		200	1			41.8
12			1.5			35.9
13			2			37.8
14			2.5			33.9
15			3			35.8
16			2	8.5		39.6
17				9.5		44.9
18				10.5		37.9
19				11.5		36.5
20				12.5		28.9
21				10.5	0.3	44.7
22					0.4	38.6
23					0.5	37.8
24					0.6	41.5
25					0.7	37.4

**Table 3:** Experiment parameters designed by using the Taguchi method and average hardness values.

No.	Travel speed (mm/min)	Current intensity (A)	Gas flow rate (l/min)	Arc length (mm)	Pulse time (s)	HRC
1	150	100	8.5	1	0.3	41.4
2	150	110	9.5	1.5	0.4	39.0
3	150	120	10.5	2	0.5	37.8
4	150	130	11.5	2.5	0.6	42.6
5	150	140	12.5	3	0.7	38.3
6	175	100	9.5	2	0.6	40.9
7	175	110	10.5	2.5	0.7	39.6
8	175	120	11.5	3	0.3	35.4
9	175	130	12.5	1	0.4	35.5
10	175	140	8.5	1.5	0.5	28.8
11	200	100	10.5	3	0.4	34.1
12	200	110	11.5	1	0.5	38.9
13	200	120	12.5	1.5	0.6	34.6
14	200	130	8.5	2	0.7	35.5
15	200	140	9.5	2.5	0.3	36.7
16	225	100	11.5	1.5	0.7	28.8
17	225	110	12.5	2	0.3	28.2
18	225	120	8.5	2.5	0.4	35.6
19	225	130	9.5	3	0.5	31.8
20	225	140	10.5	1	0.6	30.0
21	250	100	12.5	2.5	0.5	34.9
22	250	110	8.5	3	0.6	32.3
23	250	120	9.5	1	0.7	29.4
24	250	130	10.5	1.5	0.3	37.3
25	250	140	11.5	2	0.4	31.9

Before arc hardening, the steel samples are annealed to remove the residual stress and improve uniform characteristics. Notably, the arc hardening process is the self-quenching method; therefore, there is no need for further artificial cooling steps like the conventional hardening process. After hardening, the hardness measurements of the samples (S1 Fig) were performed using an HR-150A Rockwell hardness tester (Yisite, Shenzhen, China) and were shown in column 7 of Tables 2 and 3. This measuring device has a measurement tolerance of 0.5 HRC. The hardness value was measured with 10 points on the test sample; the measured values of each measuring point of the sample are shown in Tables S1 and S2. Also in these two tables, in addition to the hardness values measured at 10 points, the median values, range values, sum of squared deviation values, same variance values and standard deviation values are also included. In the range value column, the measurement range was shown from 1 to 2 HRC, which was a reliable range. Next, samples were also cut by a wire electric discharge machine (EDM) to measure the microstructure and microhardness. The microhardness was examined by Vickers hardness tester HV_0.3_ (HM101 Mitutoyo, Tokyo, Japan). This measuring method employs a square-based pyramid indenter whose opposite sides meet at the apex at an angle of 136°. Vickers microhardness was calculated by Equation 1.


$$HV=\frac{2Fsin\frac{136^{o}}{2}}{d^{2}}\;0.1\approx 0.1854\frac{F}{d^{2}}\;(N/{mm}^{2})$$ (1)

F (N): is the force applied to the diamond.

d (mm): Arithmetic mean of the two diagonals of indentations.

Microstructural analysis was performed using an Oxion OX.2153-PLM microscope (EUROMEX, Holland). Before observation, the sample is ground and polished. The sample will be sanded on sandpaper with the abrasive grit sizes 180-240-300-400, respectively. The polishing process is performed on the EcoMet 30 Buehler machine with 500 rotation cycles per minute. After polishing, the sample will be etched with a 4% Nital solution at an ambient temperature of 30 ° C. Additionally, a scanning electron microscope (SEM) called the JEOL 5410 LV (Japan) was used for observing the sample surfaces.

## Results and discussion

**Fig 2:**
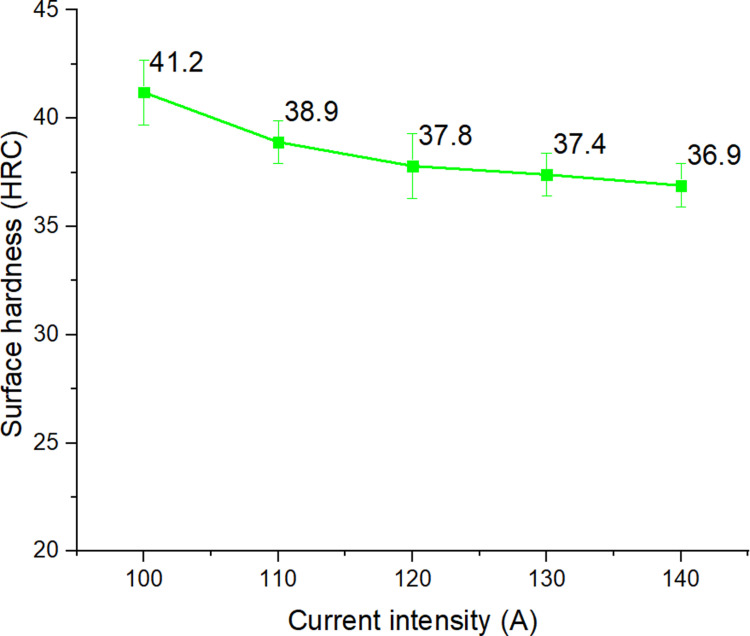
Effects of current intensity on the surface hardness of S45C steel with a curve shape.

**Fig 3:**
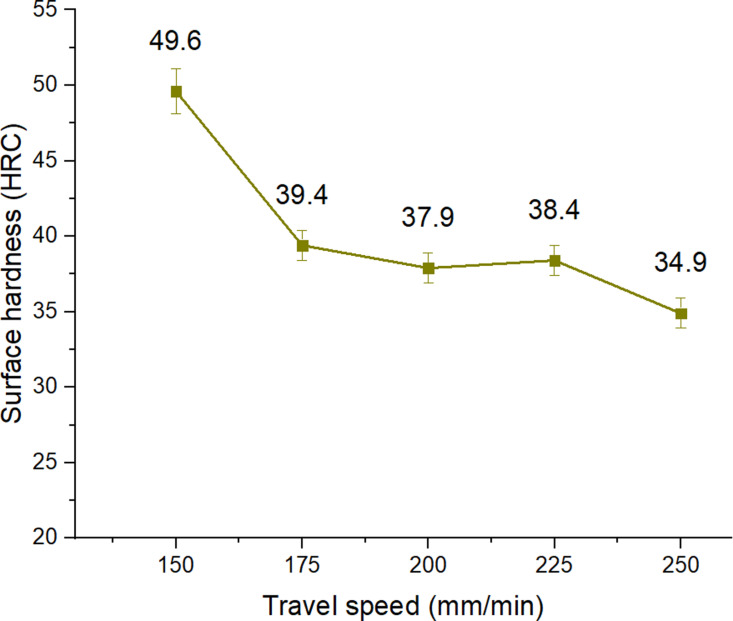
Effects of Travel speed on the surface hardness of S45C steel with a curve shape.

**Fig 4:**
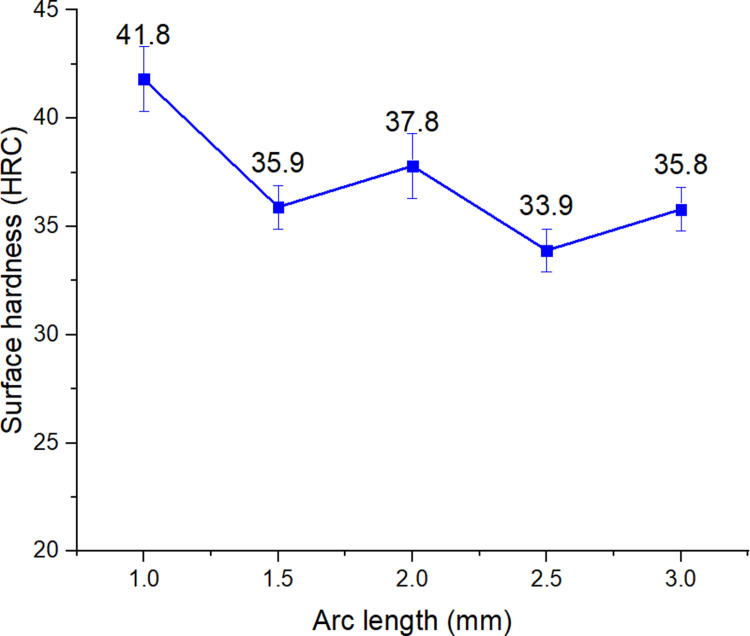
Effects of arc length on the surface hardness of S45C steel with a curve shape.

**Fig 5:**
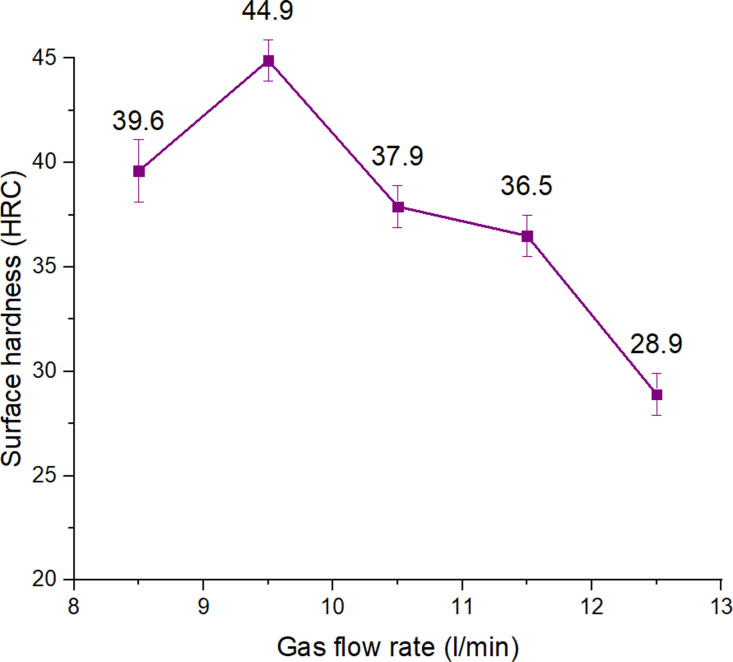
Effects of gas flow rate on the surface hardness of S45C steel with a curve shape.

**Fig 6:**
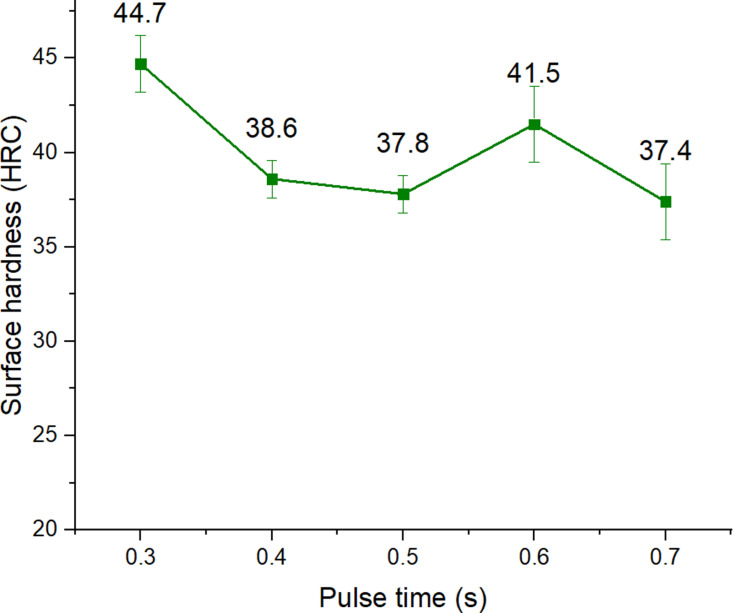
Effects of pulse time on the surface hardness of S45C steel with a curve shape.

Column 7 of Table 2 was the hardness measurement result after performing the surface quenching process by arc, with the process parameters shown in columns 2, 3, 4, 5, and 6. Figs 2 to 6 were drawn from the surface hardness measurement results after arc quenching of the corresponding samples 1–5, 6–10, 11–15, 15–20, 21–25.

### Effects of current intensity on the surface hardness

Fig 2 shows the effects of current intensity on the surface hardness of S45C steel with a curve shape. The results show that the hardness of the sample has improved significantly compared to the as-received sample (18 HRC), reaching up to 2.3 times higher values. Specifically, the hardness could reach a high value of 41.2 HRC at 100 A. Increasing the current intensity from 100 A to 140 A leads to a gradual decrease in the surface hardness from 41.2 HRC to 36.9 HRC. Overall, the hardness parameter is inversely proportional to the current intensity, which is similar to Mikheev et al. [20] study. According to the moving arc heat model of Nguyen et al. [27], it was shown that the current intensity is a factor affecting the heat generated from the TIG tip with Q = *µ.I.V* (Joule). Where *I* was the arc current intensity, *V* was the arc voltage, and *µ* was the arc efﬁciency. Therefore, when improving the current leads to a higher heat input [19], the surface can be melted and reduce the surface hardness.

### Effects of Travel speed on the surface hardness

Travel speed directly affects the heat input during cooling, microstructure, and surface hardness [19]. Therefore, studying the effect of TIG travel speed during arc cooling is very important to improve the surface hardness. Fig 3 shows the effects of Travel speed on the surface hardness of S45C steel with a curve shape. Compared to the as-received samples, applying the arc hardening at different Travel speeds also results in raising the surface hardness of the samples. In other words, arc hardening is efficient in improving the surface hardness. The surface hardness has the highest value of 49.6 HRC at the Travel speed of 150 mm/min. When gradually increasing the Travel speed to 250 mm/min, the surface hardness also mainly decreases to 34.9 HRC. The reason is that improving the travel speed could help improve the self-quenching phenomenon. This result is consistent with Mikheev et al. [20], which also indicated that the surface hardness reduces as the Travel speed increases. In other words, the heat input decreases as the travel speed increases, which is also explained in the study of Kumar et al.[19] with the formula Q_HIP_ = *µ.I.V/S* (Joule). Where *µ* was the arc efﬁciency, *I* was the arc current intensity, *U* was the arc voltage, and *S* was the Travel speed of the arc tip. Therefore, the self-cooling rate of the steel matrix surrounding the heated surface is higher.

### Effects of arc length on the surface hardness

Fig 4 shows the effects of arc length on the surface hardness of S45C steel with a curve shape. The results show that an increase in the arc length mostly leads to a slight reduction of the surface hardness. At 1 mm arc length, the surface hardness is 41.8 HRC, then it gradually reduces to 35.8 HRC when hardening at 3 mm. The reason is that the longer arc length could spread the heat input to wider areas and reduce the concentration in the local area. However, there is fluctuation in the surface hardness when increasing the arc length. The impact level of the arc length will be discussed more in the following Taguchi section.

### Effects of gas flow rate on the surface hardness

The gas flow rate during the TIG arc quenching process will significantly affect the surface quenching process by electric arc. It is directly related to the amount of heat transferred to the surface of the part. If the gas flow rate is too high, it will take away a part of the heat generated from the TIG tip, so the heat transferred to the steel surface will be lower than if it is low, so the surface may not be provided with enough heat. In addition, the gas flow rate also affects the cooling rate of the steel surface after heating. If the gas flow rate is high, the surface cooling rate will occur faster, creating more martensite phase, which will increase the hardness. The interesting thing here is that we clearly see that they both affect the input heat and the cooling rate to increase the hardness. Determining the appropriate gas flow rate is one of the factors that need to be studied in this study. Fig 5 shows the effects of gas flow rate on the surface hardness of S45C steel with a curve shape. There are two stages of the hardness pattern when increasing the gas flow rate from 8.5 l/min to 12.5 l/min. Firstly, from 8.5 to 9.5 l/min, an increase in the gas flow rate leads to an enhancement in the surface hardness from 39.6 HRC to 44.9 HRC. This can be understood as when increasing the gas flow, the rapid cooling process of the part after receiving heat from the TIG head leads to the creation of more martensite phase, leading to an increase in hardness. Then, a further increase in the gas flow rate from 9.5 l/min to 12.5 l/min results in a rapid decline of surface hardness from 44.9 HRC to 28.9 HRC. This can be explained by the fact that a large gas flow will take away some of the heat generated from the TIG tip, reducing the heat input to the quenching process. This reduction in heat input will prevent the steel surface from being heated to the appropriate temperature for the quenching process by the arc. The influence level of the gas flow rate will be discussed more in the following Taguchi section.

### Effects of pulse time on the surface hardness

Pulse time directly affects the amount of heat generated at the TIG tip [28]. The mathematical model of the 3D moving heat source in the form of a double ellipse was proposed by Nguyen et al. [27], indicating that the pulse time is an input factor to generate the amount of heat at the TIG tip. During the pulse time, a large amount of heat was generated to melt the material; during the non-pulse time, the heated area was cooled down, increasing the hardness of the steel surface [29]. Therefore, applying pulse time could achieve better heat control, reducing the overheating phenomenon. Fig 6 shows the effects of pulse time on the surface hardness of S45C steel with a curve shape. The surface hardness obtains its highest value at a pulse time of 0.3 s with 44.7 HRC. Then it gradually reduces to 37.8 HRC at 0.5 s, before rising to 41.5 HRC at 0.6 s. Finally, it reduces to 37.4 HRC at a pulse time of 0.7 s. The tendency is that when the pulse time increases, the surface hardness will decrease. This can be explained by the phenomenon of overheating [30]. When the pulse time is quite long, the heat generated from the TIG arc will be larger, which will lead to too much heat, slowing down the cooling rate of the surface quenching process, leading to less phase transformation from austenite to martesite, so the surface hardness will decrease. The impact level of the pulse time will be discussed more in the following Taguchi section.

### Taguchi results

**Fig. 7:**
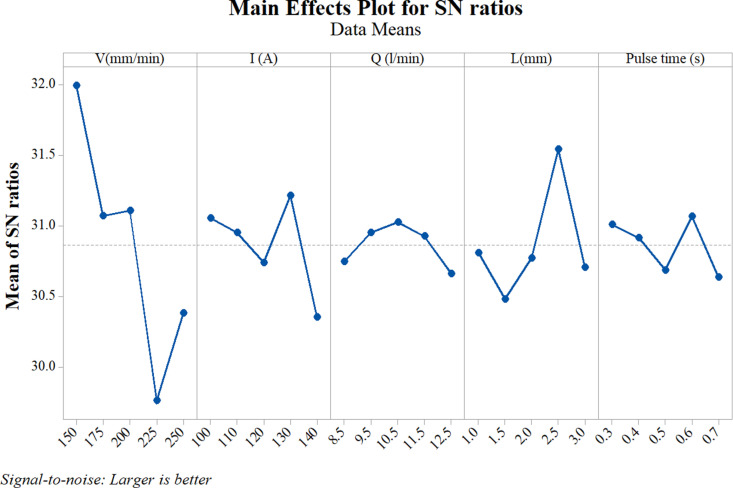
Main effects plot for SN ratios of the S45C steel hardness with a concave surface (larger is better).

Besides the traditional examination by surveying individual parameters, this study also tries to evaluate the impact ranking of these parameters. Table 4 and Fig 7 present the signal-to-noise (S/N) value of the voltage, current intensity, arc length, gas flow rate, and pulse factors that influence the surface hardness of the samples. In Table 4, the Delta value (row 7) was calculated by subtracting the smallest value from the largest value of each experimental parameter; Rank (row 8) shows the influence ranking of the experimental parameter, and a larger Delta value shows the greater influence on the surface hardness. The Travel speed factor appears to have the strongest impact level, followed by the gas flow rate and current intensity. Pulse time and arc length rank for the fourth and fifth positions, indicating their weaker impact on the surface hardness. In other words, focusing on voltage and gas flow rate could lead to better changes in the surface hardness.

**Table 4:** Evaluate results by parameters affecting S/N ratio.

Level	Travel speed	Current intensity	Arc length	Gas flow rate	Pulse time
1	31.99	31.05	30.74	30.81	31.01
2	31.07	30.95	30.95	30.48	30.92
3	31.11	30.74	31.03	30.77	30.69
4	29.76	31.21	30.92	31.54	31.06
5	30.38	30.35	30.66	30.70	30.64
Delta	2.23	0.87	0.36	1.06	0.42
Rank	1	3	5	2	4

The values of Table 4 were used to draw Fig 7. This figure shows the influence of the parameters on the surface hardness. The slope of the graph was larger, the influence was larger. The upper peaks of the graphs of each parameter represent their optimum values. For the travel speed, the optimum value is 150 mm/min; the current intensity has an optimum value of 130A; the gas flow rate has an optimum value of 10.5 l/min; the return length is 2.5 mm, and the pulse value is 0.6 s.

### Results of 3D surface plot

**Fig 8:**
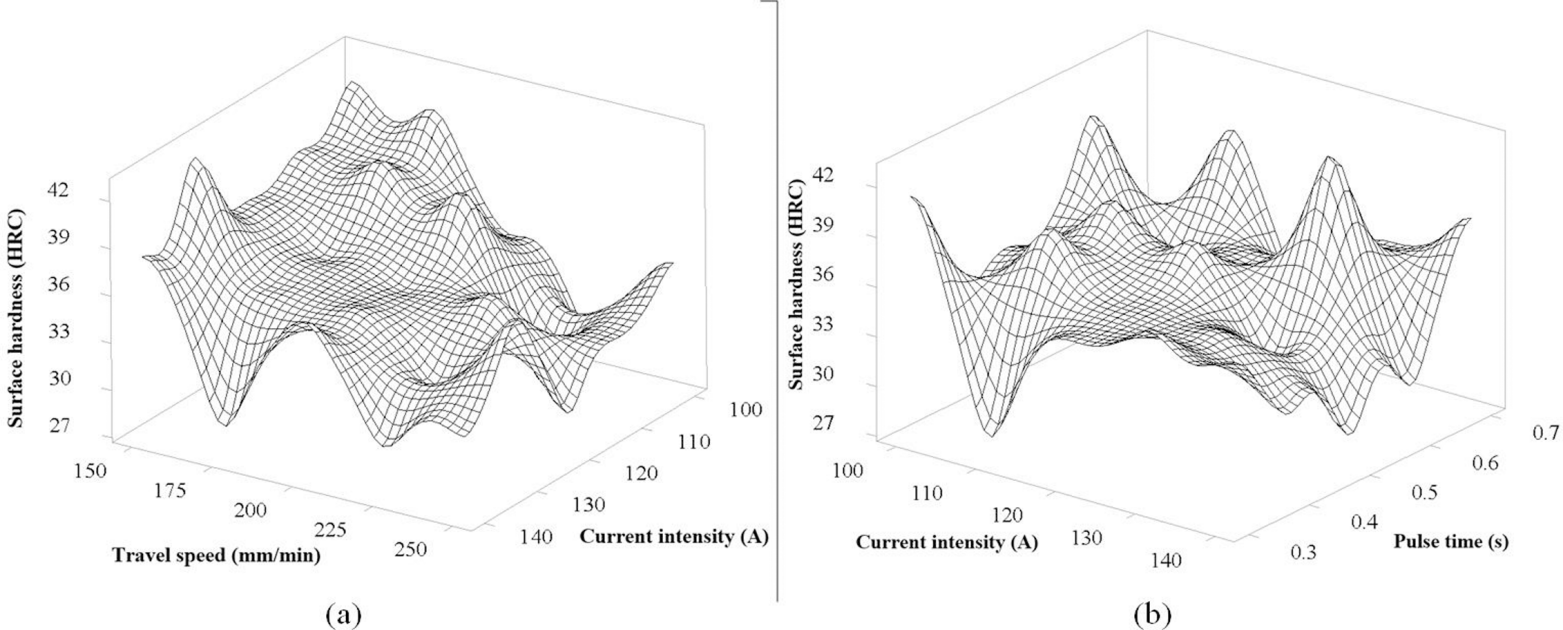
The 3D surface graph of interaction between Current intensity, Travel speed and Pulse time for the surface hardness of S45C steel after electric arc quenching: (a) The 3D surface graph of Surface hardness versus Current intensity and Travel speed, (b) The 3D surface graph of surface hardness versus Current intensity and Pulse time.

The 3D surface graph represents the relationship between Surface hardness, Current intensity, and Travel speed, as illustrated in Fig 8a. In this figure, when considering the direction of change of the travel speed of the TIg head, the trend of decreasing surface hardness is clear when the travel speed increases, represented by the tilt direction of the model. This is consistent with the theory of heat input from the TIG weld pool transmitted to the steel surface of the studies [22,26,28], where increasing the travel speed will reduce the input heat. For the current intensity, the figure also shows a clear trend that increasing the current intensity increases, the surface hardness tends to decrease. This is consistent with the theory of the weld pool, where increasing the current intensity will increase the input heat, slowing down the rapid cooling process, and decrease surface hardness. In general, these two factors have strong interactions with each other, so the graph has a clear influence trend. The position with the highest hardness in the figure is obtained when the current intensity is at 130 A and the moving speed is at 150 mm/min. Fig 8b shows the correlation between Current intensity and Pulse time to surface hardness. In general, the graph tends to be stable in the range of 0.3 s to 0.5 s for pulse time when the hardness tends to decrease as the current intensity increases, which has the same result as Fig 8a. From 0.6 s to 0.7 s, the hardness has an unclear change, which is explained by the pulse time being too long, causing overheating [29] and causing instability in the hardness evaluation experiment. The maximum hardness obtained for the interaction of these two factors is the current intensity at 130 A and the pulse time at 0.6 s, corresponding to the highest peak of the graph.

**Fig 9:**
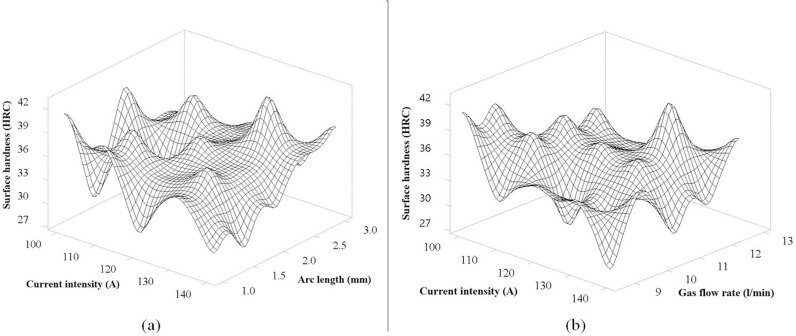
The 3D surface graph of interaction between Current intensity, Arc length and Gas flow rate for the surface hardness of S45C steel after electric arc quenching: (a) The 3D surface graph of Surface hardness versus Current intensity and Arc length, (b) The 3D surface graph of surface hardness versus Current intensity and Gas flow rate.

The 3D surface graph represents the relationship between Surface hardness, Current intensity, and Arc length, as illustrated in Fig 9a. In the arc length range from 1 mm to 2 mm, the graph of the figure shows more clarity and stability with the same rule as in Fig 8 that when the current intensity increases, the surface hardness tends to decrease due to the increased input heat, causing the rapid cooling process of the sample surface to decrease. From about 2.5 mm to 3 mm, the graph of this figure shows instability when many peaks appear, and there is no clear rule. This can be explained by the large arc length, leading to the heat transfer process from the TIG head being strongly affected by environmental factors, and is explained by studies [22, 26, 28, 30] as the arc efficiency (µ). Fig 9b shows the correlation between Current intensity and Gas flow rate to surface hardness. For this figure, the surface graph shows a stable range and a decreasing hardness rule when the gas flow rate increases from 9.5 l/min to 12.5 l/min, corresponding to the decreasing slope of the graph. This is explained that when the gas flow rate is too large, it will take away the heat generated from the TIG tip and transfer it to the shielding gas, thereby reducing the heat input in the surface quenching. In addition, the figure also shows a similar rule as Figs 8 and 9a, that when the current intensity increases, the surface hardness will decrease. The peak of the figure corresponds to the optimal hardness value achieved when the current intensity is at 130 A and the gas flow rate is at 12.5 l/min.

**Fig 10:**
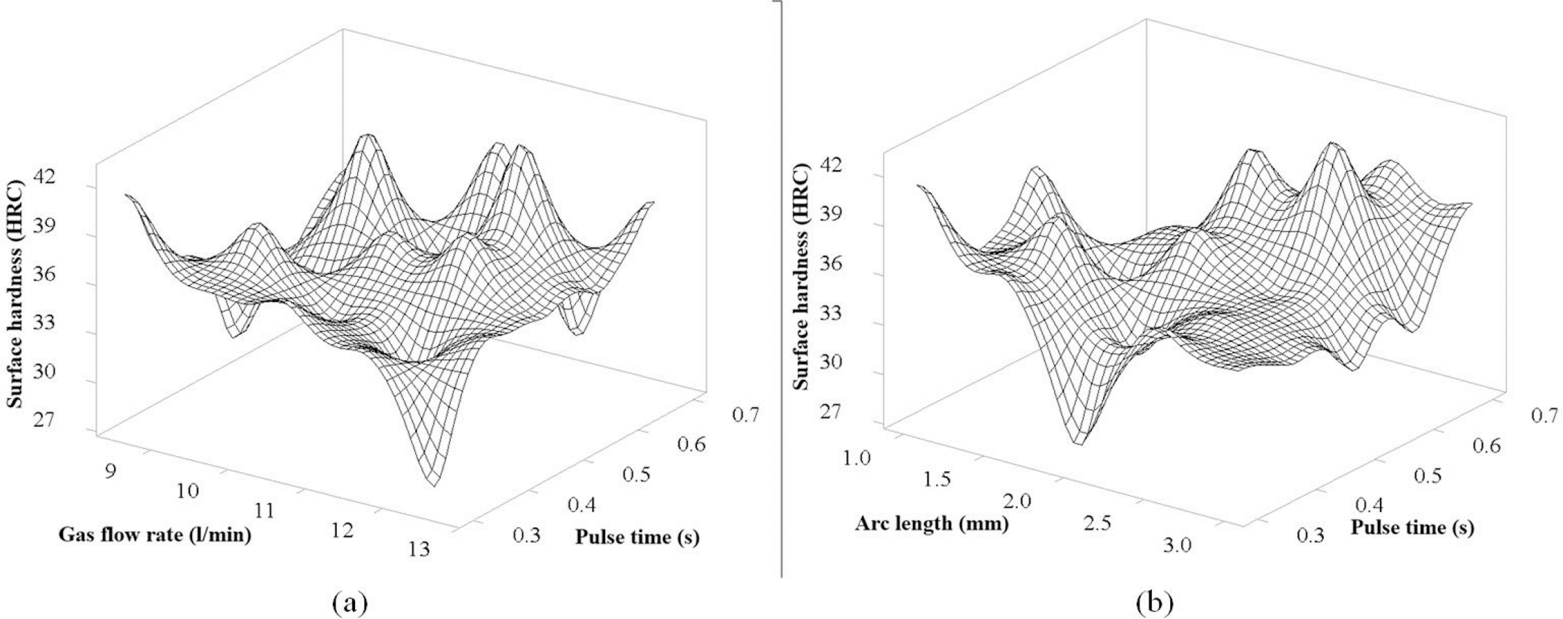
The 3D surface graph of interaction between Pulse time, Arc length and Gas flow rate for the surface hardness of S45C steel after electric arc quenching: (a) The 3D surface graph of Surface hardness versus Pulse time and Arc length, (b) The 3D surface graph of surface hardness versus Pulse time and Gas flow rate.

Fig 10a shows the correlation between Pulse time and Gas flow rate to surface hardness. In general, the graph shows the hardness stability at the pulse time from 0.3s to 0.5s. In this range, there is no clear interaction according to a certain rule. However, when the pulse time fluctuates from 0.6s to 0.7s, there are 3 peaks on the graph corresponding to the gas flow levels of 9.5 l/min and 0.6s pulse time; at 10.5 l/min and 0.7 s pulse time; at 11.5 l/min and 0.6 s pulse time. This shows that when the pulse time is prolonged, the heat generated will be large, leading to instability in controlling the surface quenching process of S45C steel. Fig 10b shows the interaction of arc length with pulse time on surface hardness. In the range of 1.5 mm to 2.5 mm arc length, as pulse time increases, the graph shows a certain rule that surface hardness increases. This is an interesting interaction, as pulse time increases, the heat input increases correspondingly, resulting in higher surface hardness. This is the ideal arc length range when considering the interaction with pulse time, this range is also consistent with the interaction analyzed in Fig 9a.

**Fig 11:**
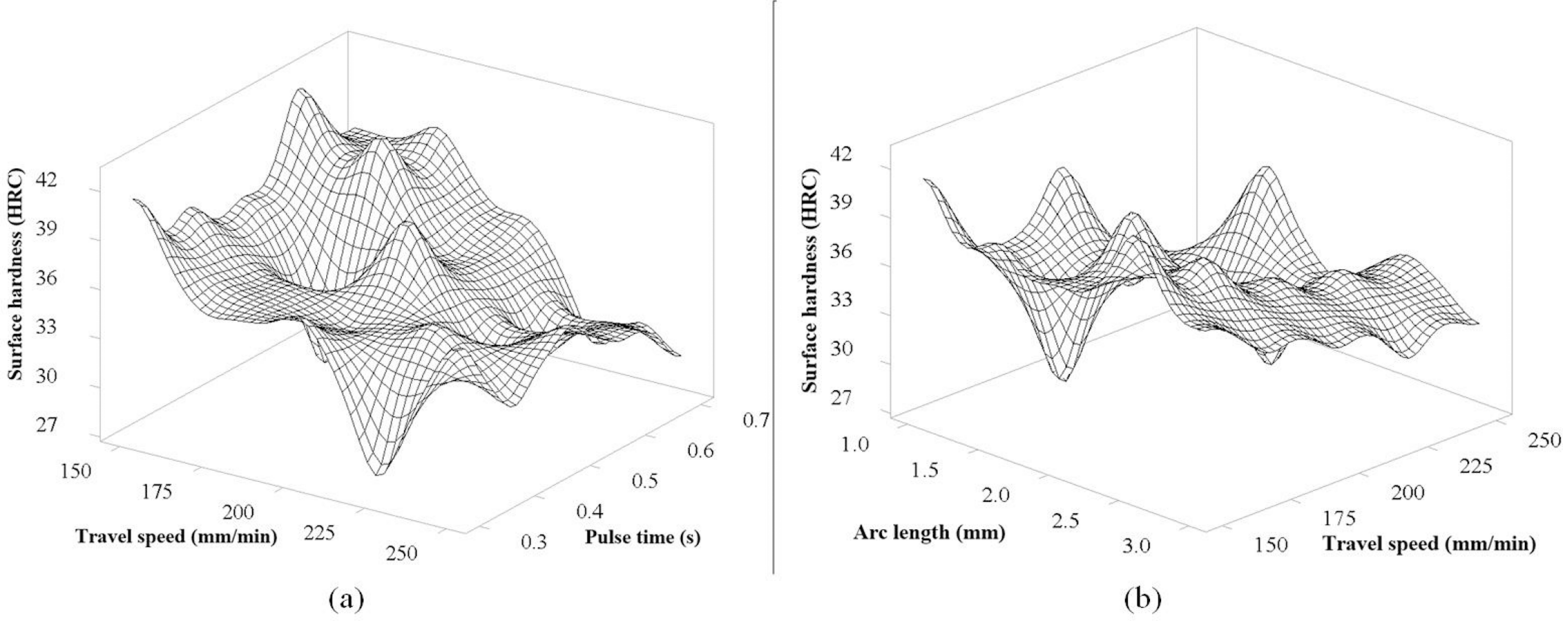
The 3D surface graph of interaction between Pulse time, Arc length and Travel speed for the surface hardness of S45C steel after electric arc quenching: (a) The 3D surface graph of Surface hardness versus Pulse time and Travel speed, (b) The 3D surface graph of surface hardness versus Travel speed and Arc length.

Fig 11a shows the interaction between the pulse time and the travel speed of the TIG head. The interesting thing here is that the graph clearly shows the interaction between the two variables and the surface hardness. The graph tends to decrease the hardness as the travel speed increases and the pulse time increases. This is consistent with Fig 8a. When the travel speed increases, the heat input to the surface will decrease due to the fast travel, so the heat received by the S45C steel surface will be lower [22, 26, 28], and there will not be enough heat to completely transform the ferrite and pearlite phases to the austenite phase. Therefore, after rapid cooling, the amount of martenite produced is less, and the surface hardness will be lower. For the pulse time, on the contrary, when the pulse time is increased, the heat input will increase, causing overheating [30], thus slowing down the surface cooling process, and less martensite phase will be produced due to the phase transformation from austenite to pearlite phase. The highest peak of the surface graph corresponding to the highest surface hardness is at the point where the travel speed is 150 mm/min and the pulse time is 0.6 s. Fig 11b shows the interaction between the arc length and the travel speed of the TIG head. Similarly, the same rule as Figs 11a and 8a, as increasing the travel speed, the surface hardness decreases due to the decrease in heat input. In addition, the graph also shows that in the range of 1.5 mm to 2.5 mm for the arc length.

**Fig 12:**
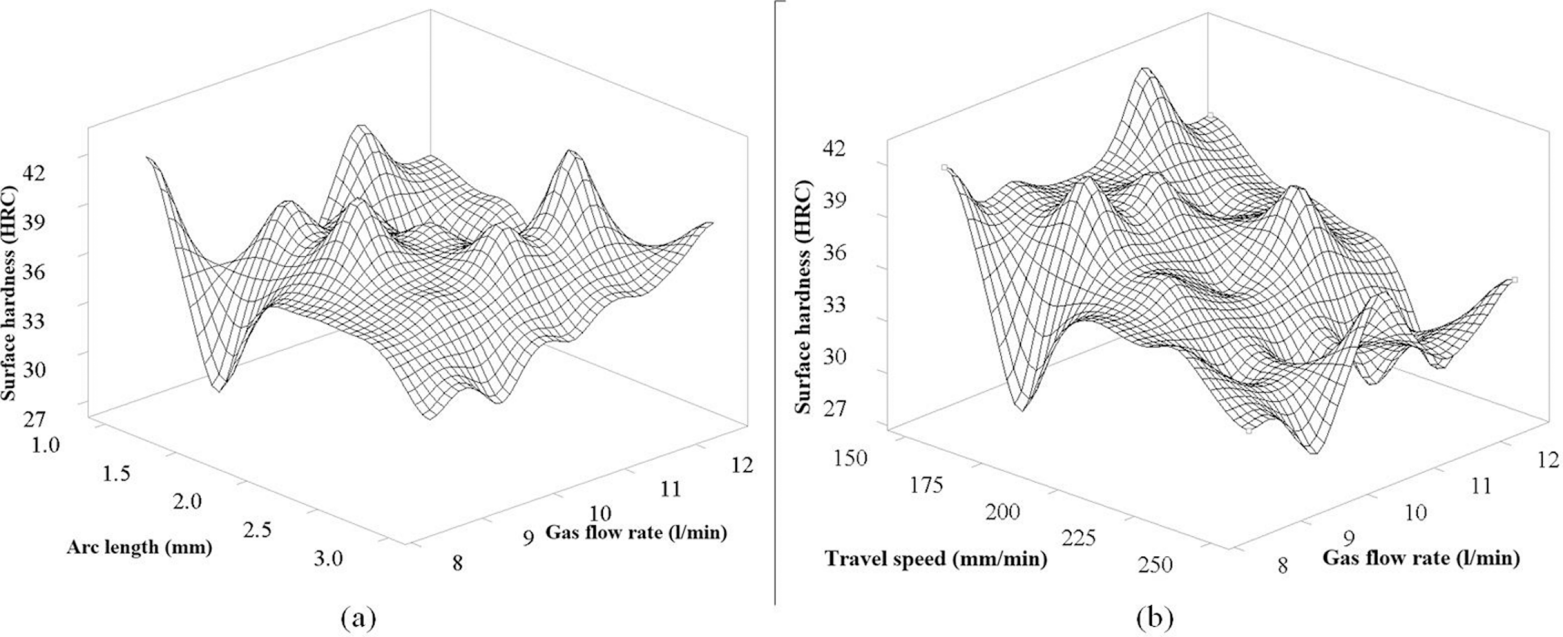
The 3D surface graph of interaction between Gas flow rate, Arc length and Travel speed for the surface hardness of S45C steel after electric arc quenching: (a) The 3D surface graph of Surface hardness versus Arc length and Gas flow rate, (b) The 3D surface graph of surface hardness versus Travel speed and Gas flow rate.

Fig 12a shows the interaction between the arc length and the gas flow rate. The graph of this figure shows the stability of hardness in the range of 1.5 mm to 2.5 mm for the arc length and 9.5 l/min to 11.5 l/min for the gas flow rate. Interestingly, both parameters of the arc efficiency are. The optimum value of the interaction of the two parameters is at the high peak of the graph at 2.5 mm for the arc length and 12.5 l/min for the gas flow rate. Fig 12b shows the interaction between the travel speed and the gas flow rate. Looking at the graph of the figure; we can see the same rule as in Figs 8a, 11a, 11b; when increasing the travel speed, the hardness surface decreases, corresponding to the slope of the circle. Similarly, when the gas flow rate increases, the hardness surface decreases. Interestingly, these two parameters are inversely proportional to the input heat, so the slope of this figure is most obvious. Also, according to this figure, the travel speed affects more than the flow when the slope is larger in the direction of the decreasing travel speed than the slope in the direction of the gas flow rate. The optimal value of hardness surface obtained corresponding to the peak of the graph is achieved at 150 mm/min for the travel speed and 11.5 l/min for the gas flow rate.

In general, through the analysis of the interaction graph between the process parameters of quenching the surface of S45C steel by the arc energy, it can be seen that the stable range of the arc length is from 1.5 mm to 2.5 mm. For the pulse time, the longer the pulse time, the stronger the impact on the heat generated from the TIG head. The ideal control range is from 0.3s to 0.6s. The increased current intensity will affect the heat generated from the TIG head and directly affect the heat input to the surface quenching process. For the travel speed, this is the parameter that directly and most affects the remaining parameters. The most ideal value is at 150 mm/min, and the surface hardness tends to decrease as the travel speed increases. Finally, the gas flow rate is a factor that affects the amount of heat lost by the TIG head. If the gas flow rate is too high, it will not be good when considering the surface quenching process due to the loss of energy. In addition, it also affects the economy when the large gas flow rate directly affects the operating cost of the quenching process.

### Microhardness and microstructure of the hardened sample

**Fig 13:**
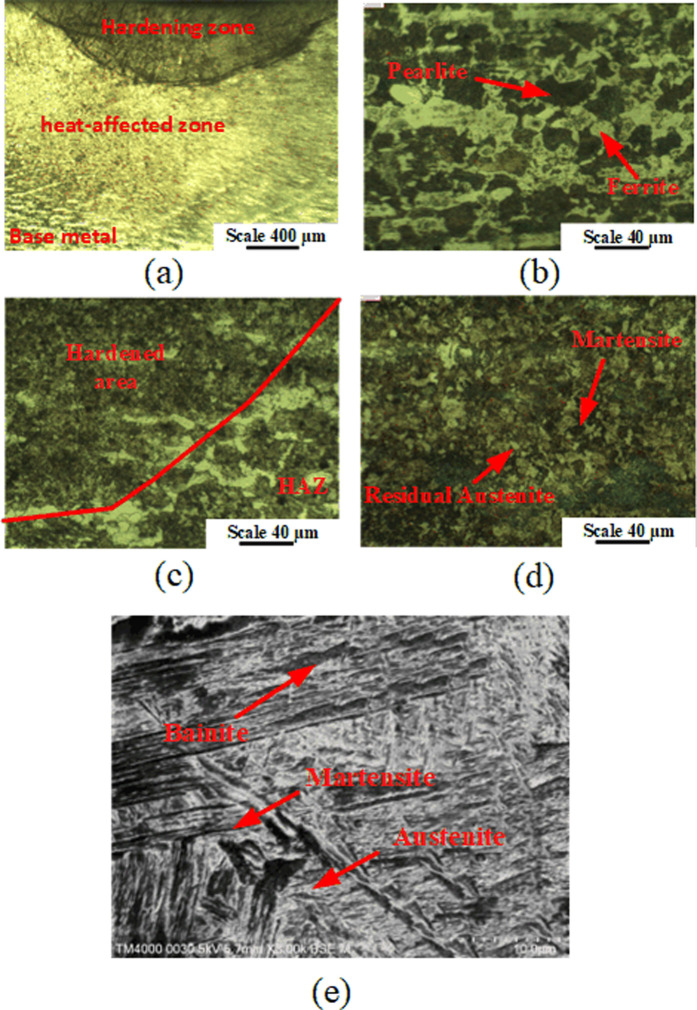
Structure of sample No. 6: (a) macrostructure (S2 Fig), (b) base metal (S3 Fig), (c) heat-affected zone, (d) hardening area (S4 Fig), and (e) hardened area under SEM (S5 Fig).

Fig 13 shows the structure of sample No. 6 after arc quenching. There are two areas with dark and bright colors, corresponding to the hardening area and the matrix, as shown in Fig 13(a). The hardening has a curved shape, presenting the heat transfer direction from the TIG source. Fig 13(b) shows the base metal consists of ferrite and pearlite, which is the original microstructure of the medium-carbon steel [31]. This structure provides high toughness for the core of the arc quenching sample. Fig 13(c) shows the HAZ, which is the transformation zone from the base metal to the hardened area. This zone consists of a bainite phase with a brown color and a ferrite phase with a brighter color, which is consistent with Kumar et al. [22] report. This zone has a relatively high hardness value and creates continuity for the extremely hard and soft areas. The microstructure of the hardening with a high-hardness value area is shown in Figs 13(d)-(e). Fig 13(d) indicates the presence of needle-shaped martensite with dark color and residual austenite with brighter color phases. With higher magnification under SEM microscope toward the dark color, Fig 13(e) shows the presence of martensite, bainite, and residual austenite. Generally, the hardening area consists of martensite, bainite, residual austenite, and ferrite phases [21,22]. The variety of these phases is due to the rapid heating and then rapid cooling rate and the high differences in cooling rate between different depths.

**Fig 14:**
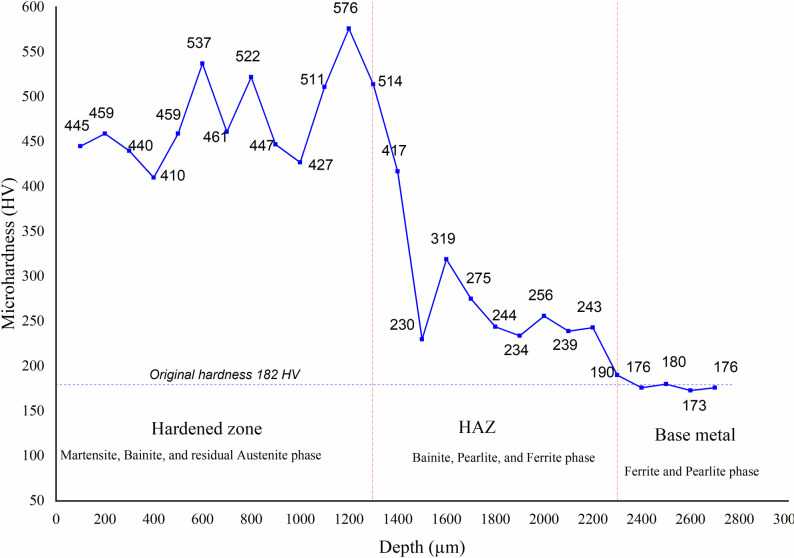
Microhardness vs. depth diagram of sample No. 6 ( S6 Fig).

Fig 14 shows the microhardness vs. depth of sample No. 6. after arc quenching. The microhardness diagram could be divided into 4 stages: improving, rapid dropping, slow dropping, and stable. In the improving stage (the stage where the surface hardness was significantly increased compared to the untreated hardness), the hardness mainly increases from 445 HV to the highest value of 576 HV, corresponding to the distance from the surface to 1200 μm depth. This 1200 μm case hardening depth is compatible with the laser hardening and electron beam hardening techniques, which are normally 100 μm to 2500 μm [32, 33]. However, the set-up cost of arc hardening is greatly lower than the laser or electron beam hardening techniques. The reason for this improvement is the reduction of the molten ratio when the depth increases; therefore, the effectiveness of the arc hardening is improved. Moreover, according to Safonov et al. [19] study, in the improving stage, the residual austenite phase content is reduced when the case depth increases, leading to the rise of the martensite phase. Therefore, the microhardness is improved in this stage. Then, it drops drastically to 230 HV at 1500 μm depth in the rapid dropping stage (the stage where the hardness value of the sample decreases rapidly), pointing out the limitation of the case hardening depth. In this stage, the slope ratio (hardness difference/depth difference) of this study at 1153 ((576 HV_0.3_-230 HV_0.3_)/(1.5 mm - 1.2 mm)) has a smaller ratio than that of the study [33] ranging from 3500-1750. This shows that the stability of this method is better than that of the laser method due to the larger and faster energy supply of the electric arc. From 1600 μm to 2300 μm depth, which corresponds to the slow-dropping stage, the microhardness slightly reduces from 319 HV to 190 HV. Finally, at the stable state, the hardness mostly oscillates around 180 HV, corresponding to the base metal where the heat does not impact its microstructure.

## Conclusion

This study examines the effects of arc length, current intensity, travel speed, gas flow rate, and pulse time on surface hardness to better understand the arc quenching of S45C steel with a curve shape. Among the significant findings that could be disclosed are:

˗ With the traditional examination method, an increase in the current intensity, Travel speed, and arc length leads to a decrease in the surface hardness. Changing the gas flow rate and pulse time has a fluctuating relationship with the surface hardness.
˗ The Travel speed factor appears to have the most significant impact, followed by the gas flow rate and current intensity. Pulse time and arc length rank fourth and fifth, respectively, indicating a smaller impact on surface hardness.
˗ The microhardness diagram can be classified into four stages: improving, rapid dropping, moderate dropping, and stable. The hardness reached a maximum of 576 HV, which corresponded to a case depth of 1200 μm.
˗ The arc-hardened sample’s structure consists of base metal, HAZ, and hardening zones. The base metal consists of ferrite and pearlite, which is the original microstructure of the medium-carbon steel. The HAZ consists of a bainite phase with a brown color and a ferrite phase with a brighter color. The hardening with a high-hardness value area consists of martensite, bainite, residual austenite, and ferrite phases. Rapid heating and cooling rates, as well as significant variations in cooling rates across depths, are the causes of these phases’ diversity. The study’s findings could help us better understand and apply arc-hardening technologies in the industry.
˗ The 3D surface graph shows that the ideal stable arc length range in interaction with other parameters is from 1.5 to 2.5mm. In addition, the ideal control range is from 0.3 s to 0.6 s for pulse time. Current intensity and travel speed are the two strongest parameters of the surface quenching process. As shown on the graph, the travel speed leads to a decrease in hardness. Similar to the current intensity, when increasing, the hardness decreases due to overheating.

These findings provide valuable insights for industries seeking cost-effective alternatives compared to laser or electron beam hardening, particularly in applications requiring enhanced wear resistance at lower processing costs.

In addition to the good results of the research achieved, the group of authors faced many challenges during the experimental process. We found that establishing the connection between the temperature from the TIG arc and the heat obtained at the surface of S45C steel still needed to be clarified. The measurement also showed certain error ranges due to the effects of the external environment during the experiment.

The research presented in this paper will be complemented in the future by focusing on the evaluation of the wear resistance and fatigue life of arc-hardened specimens under actual operating conditions. In addition, exploring the effects of different shielding gases on hardness and microstructure can further optimize the hardening process. Furthermore, the study will evaluate the effectiveness of the wear resistance and fatigue life of the method compared to other methods with similar levels, such as laser surface hardening, induction hardening, electron beam hardening, etc,. under specific operating conditions.

## Supporting information

S1 Fig Experimental samples were obtained after performing the surface quenching process on concave S45C steel. The HR-150A Rockwell hardness tester measured these samples, and the results are shown in S1 and S2 Tables.
(TIF)

S2 Fig Overview of the quenched area. After quenching, the samples were polished and etched with 4% Nital solution. The microstructure of the quenched samples were obtained by the optical microscope named Oxion OX.2153-PLM EUROMEX, Holland.
(TIF)

S3 Fig The original microstructure of the S45C steel. The base metal consists of ferrite and pearlite phases, which were obtained by the optical microscope named Oxion OX. 2153- PLM EUROMEX, Holland.
(TIF)

S4 Fig Martensite and residual austenite structures of the hardening zone. They were obtained by the optical microscope named Oxion OX.2153-PLM EUROMEX, Holland.
(TIF)

S5 Fig Martensite, Bainite, and residual Austenite phase of the hardening zone. It was also observed via a scanning electron microscope (SEM) named JEOL 5410 LV, Japan.
(TIF)

S6 Fig The microhardness measurement positions of sample 6. Hardness measurement positions were marked with red squares and measurements were conducted on the Vickers hardness tester.
(TIF)

S1 Table Surface hardness measurement results of S45C steel of 25 experimental samples according to the traditional univariate method with experimental parameters according to Table 1. In this table, each test sample (S1 Fig) was measured at 10 points, and the hardness value of the sample was taken as the average of the values of these 10 points. In addition, there are statistical results of range, standard deviation, and median.
(XLSX)

S2 Table Surface hardness measurement results of S45C steel of 25 experimental samples according to the Taguchi method with experimental parameters according to Table 2. In this table, each test sample (S1 Fig) was measured at 10 points, and the hardness value of the sample was taken as the average of the values of these 10 points. In addition, there are statistical results of range, standard deviation, and median.
(XLSX)
