# Supplementary figures and images for: Impact of arc quenching parameters on surface hardness and microstructure of S45C steel with concave surfaces
Source: PLoS One. 2025 Jun 2;20(6):e0324922. doi: 10.1371/journal.pone.0324922 (PMC12129324; doi:10.1371/journal.pone.0324922)

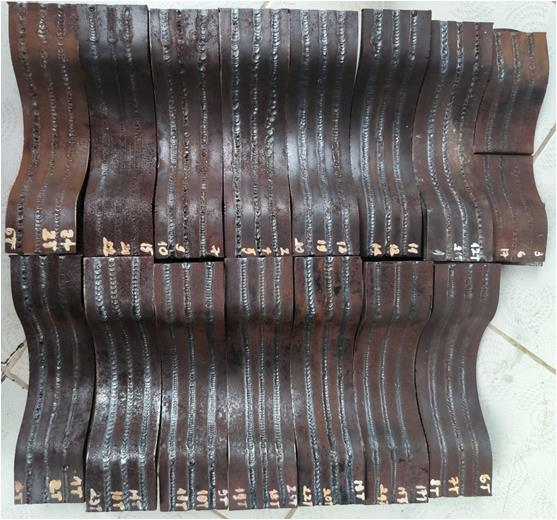

Supplement: S1 Fig — The HR-150A Rockwell hardness tester measured these samples, and the results are shown in S1 and S2 Tables. (TIF) [file pone.0324922.s001.tif]

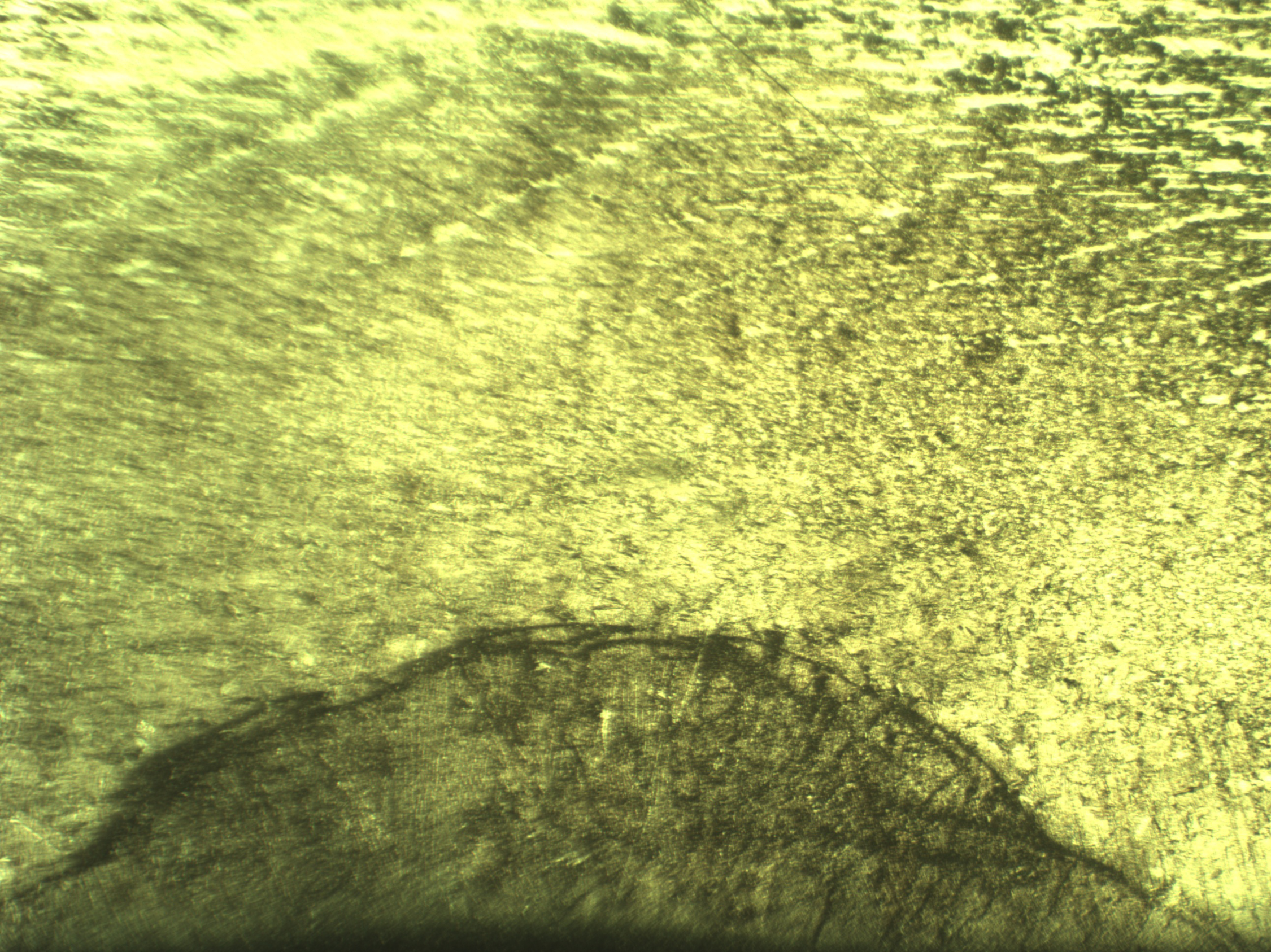

Supplement: S2 Fig — After quenching, the samples were polished and etched with 4% Nital solution. The microstructure of the quenched samples were obtained by the optical microscope named Oxion OX.2153-PLM EUROMEX, Holland. (TIF) [file pone.0324922.s002.tif]

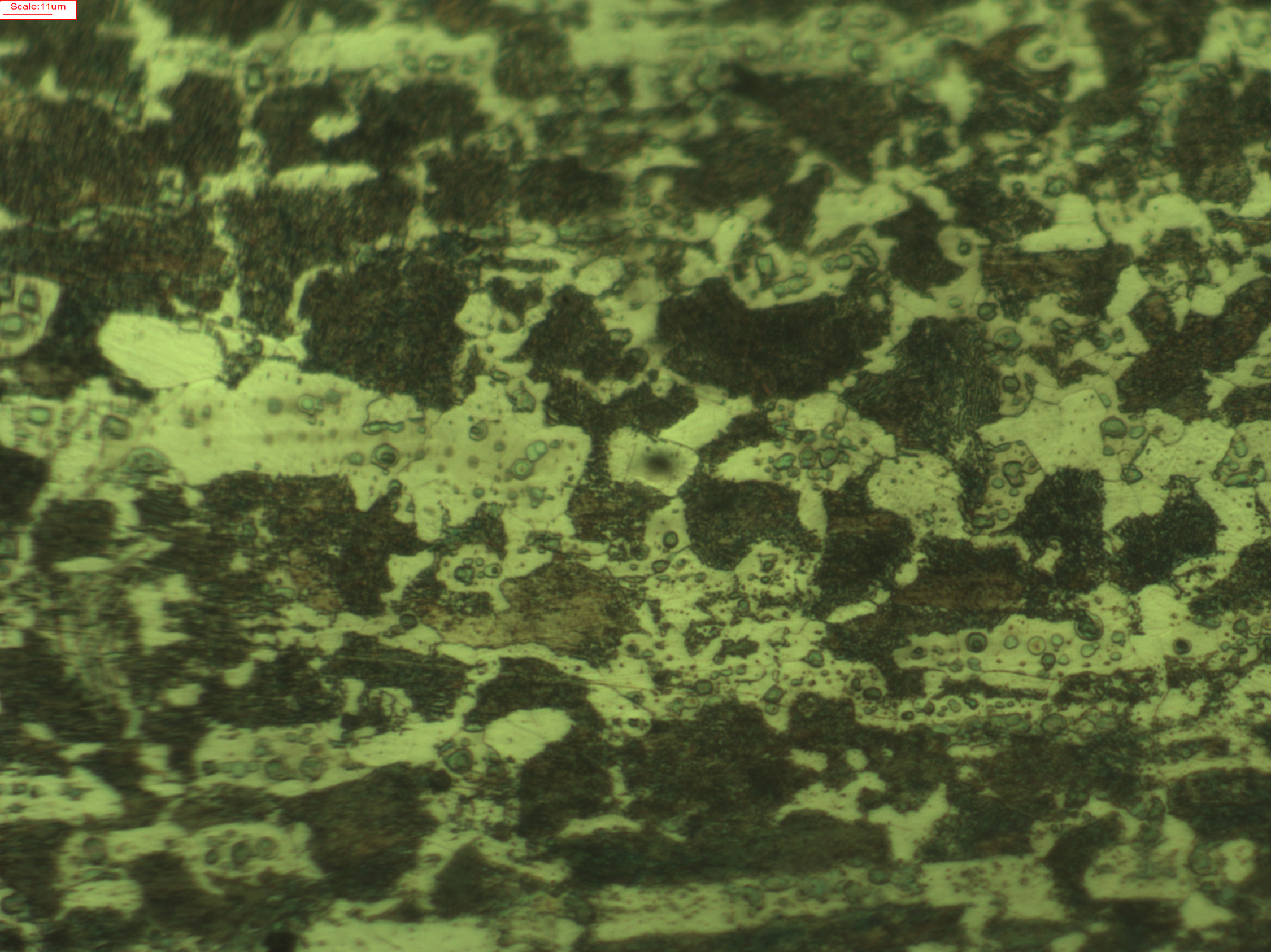

Supplement: S3 Fig — The base metal consists of ferrite and pearlite phases, which were obtained by the optical microscope named Oxion OX. 2153- PLM EUROMEX, Holland. (TIF) [file pone.0324922.s003.tif]

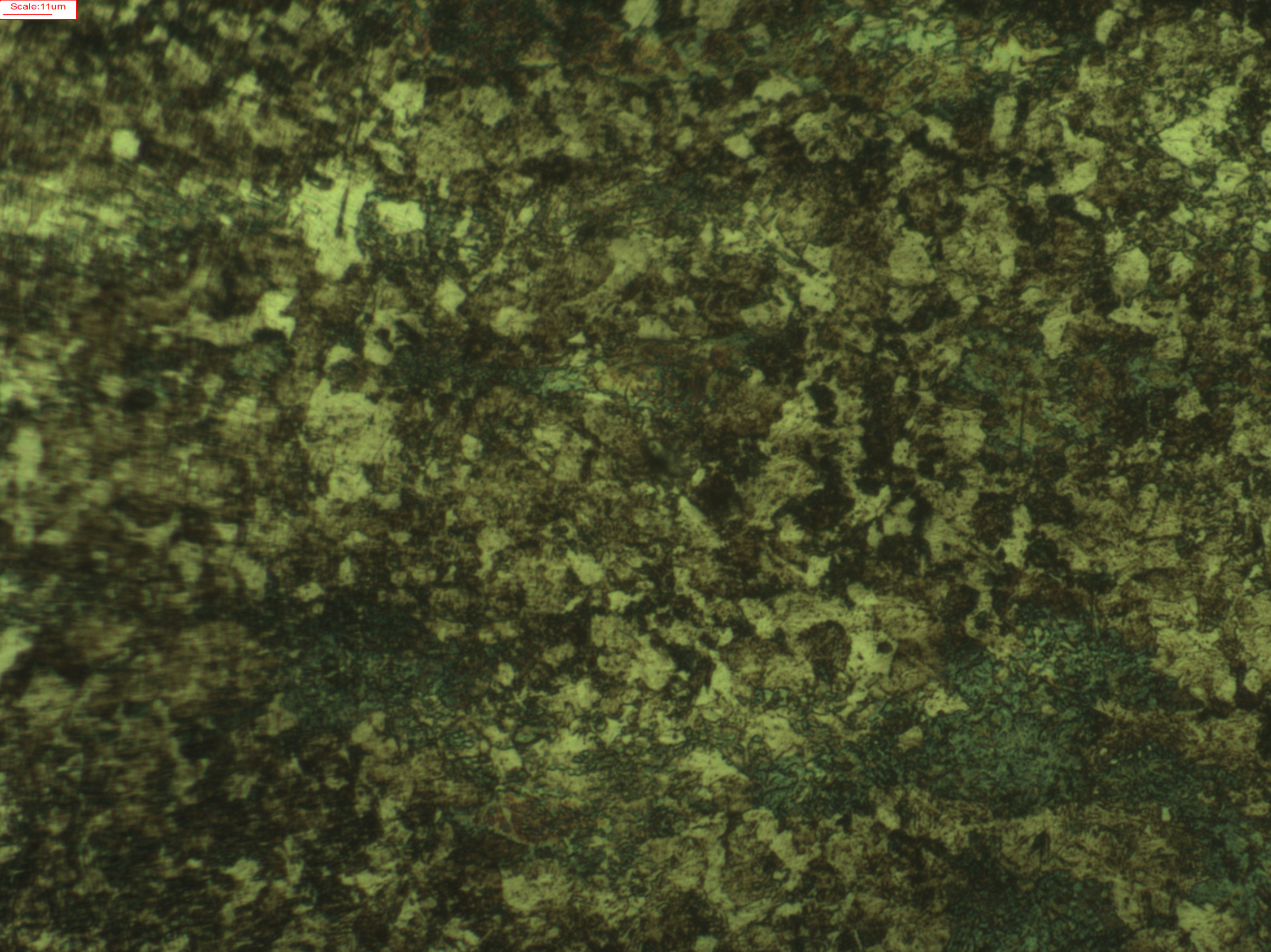

Supplement: S4 Fig — They were obtained by the optical microscope named Oxion OX.2153-PLM EUROMEX, Holland. (TIF) [file pone.0324922.s004.tif]

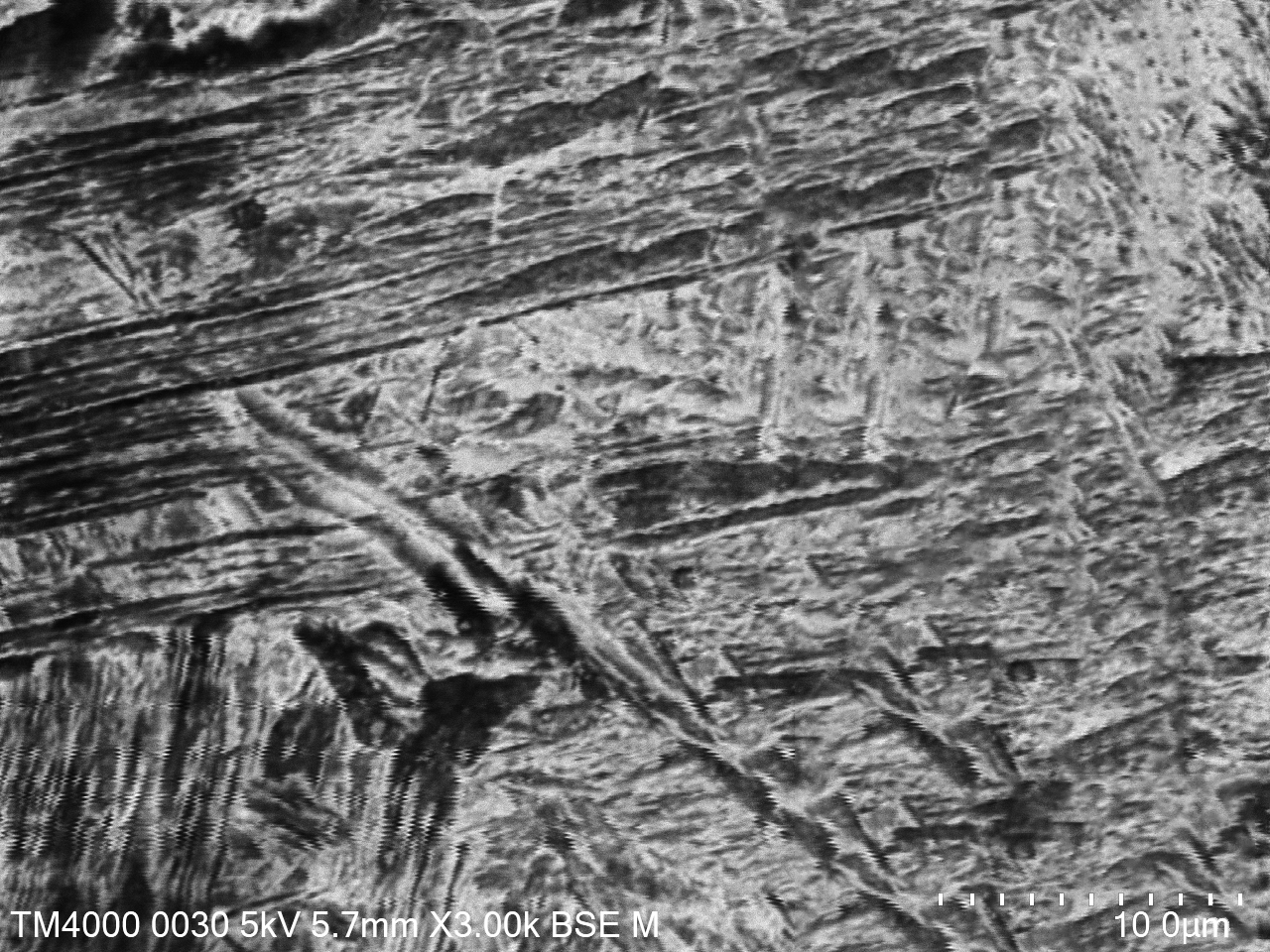

Supplement: S5 Fig — It was also observed via a scanning electron microscope (SEM) named JEOL 5410 LV, Japan. (TIF) [file pone.0324922.s005.tif]

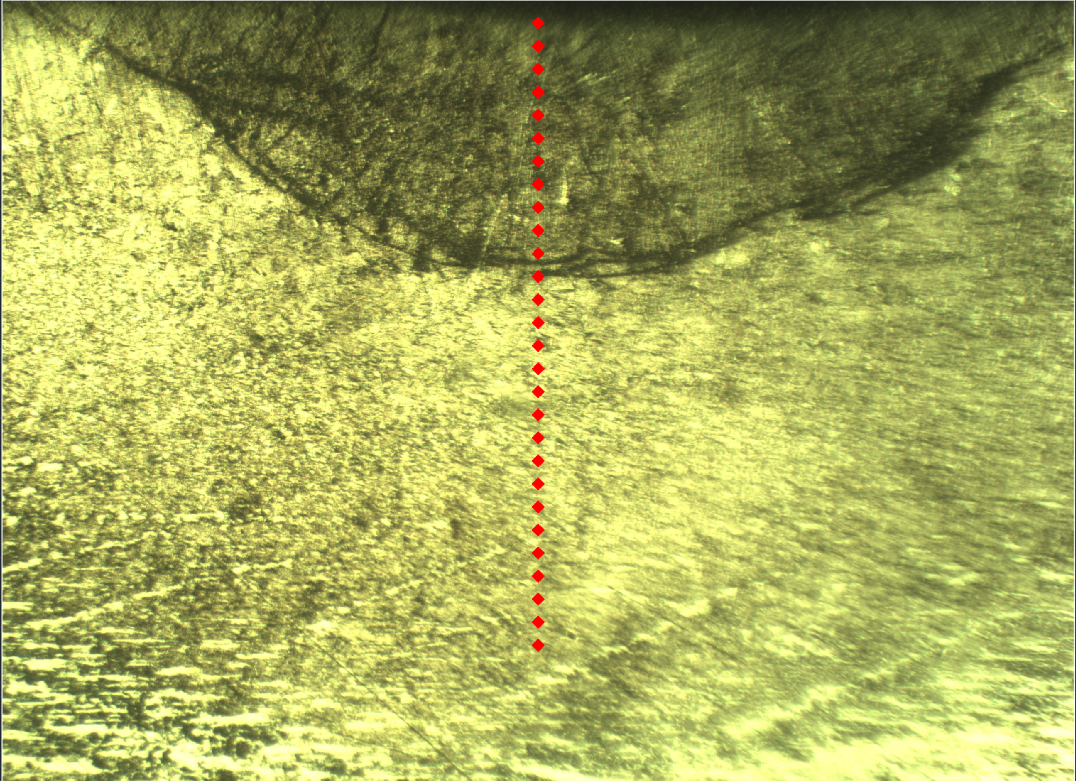

Supplement: S6 Fig — Hardness measurement positions were marked with red squares and measurements were conducted on the Vickers hardness tester. (TIF) [file pone.0324922.s006.tif]
